# Management of patients with BRCA mutation from the point of view of a breast surgeon

**DOI:** 10.1016/j.amsu.2021.102311

**Published:** 2021-04-16

**Authors:** M.L. Riis

**Affiliations:** Department of Oncology, Section of Breast- and Endocrine Surgery, Oslo University Hospital, Oslo, Norway

**Keywords:** Breast cancer, BRCA mutation, Genetically caused breast cancer, Surgery

## Abstract

Germ-line mutation in BRCA (BReast CAncer gene) 1 or BRCA2 are found in 3–4% of all women with breast cancer. These patients have a significant increased risk of breast and ovarian cancer. They are often younger when diagnosed with the mutation, and the possible breast cancer they get is often aggressive with inferior outcome. There are risk reducing strategies, and the most powerful strategy is risk reducing surgery, both risk reducing bilateral mastectomy (RRM) and risk reducing bilateral salpino-oophorectomy (PBSO). This review is meant to address breast surgery in patients with germline BRCA mutation. The guidelines and techniques applied is under continuous change and it is important for the clinicians to be well informed to provide the patient with the information needed for them to make an informed decision on what risk strategy to choose.

## Highlights

• Patients with germ-line mutation in BRCA1 or BRCA2 have a significant increased risk of breast and ovarian cancer.
• There are different risk reducing strategies and the most powerful strategy is risk-reducing surgery, both risk reducing bilateral mastectomy and risk reducing bilateral salpingo-oophorectomy.
• Guidelines and techniques for the risk reducing surgery of the breast are under continuous change and improvement.
• Breast conserving therapy is not associated with worse survival and is a good option for a BRCA mutation carrier diagnosed with breast cancer. Risk-reducing mastectomy can be performed in a later setting.
• The management of BRCA mutation carriers, both affected and unaffected, should be performed in a multidisciplinary team. Physicians need to be systematically educated and updated on the most recent literature.

## 1 Individuals with increased risk of breast cancer

### 1.1 BRCA mutation and breast cancer risk

Breast cancer is still the most common cancer among women worldwide, and the second leading cause of cancer related death [1]. There is a significant increased risk of breast cancer in patients with mutation in a high-penetrance breast cancer susceptibility gene. The magnitude differs between the genes involved. Germ-line mutation in BRCA1 or BRCA2 are found in 3–4% of all women with breast cancer ([2,3]). BRCA1 is located on the long arm of chromosome 17, cytogenetic location 17q21 [4]. BRCA2 is located on the long arm of chromosome 13, cytogenetic location 13q12.3 [5]. Mutations in BRCA1 and 2 are inherited in an autosomal dominant fashion. On the cellular level they act recessively as tumor suppressor genes involved in homologous repair of double-stranded DNA breaks [6]. Large rearrangements and deletions in the genes can also alter the function resulting in the same increased risk of breast and ovarian cancer [7]. The cumulative breast cancer risk to age 80 years is 72% (95% CI, 65%–79%) for female with BRCA1 mutation and 69% (95% CI, 61%–77%) for female with BRCA2 mutation. The former has an increased risk of breast cancer until 30–40 years, while the latter the same increased risk until 40–50 years. From then on, the incidence rate is similar and constant at 20–30 per 1000 person-years until age 80 [8]. A combined analysis of 22 studies showed similar cumulative incidence in women with the two mutations separately [9]. They do however stress that the published estimates for penetrance vary according to how the study population is selected. Studies based on families with multiple cases generally have a higher penetrance than those based on unselected cases [9]. The 10-year cumulative risk of contralateral breast cancer is 5.1% for non-carriers, 21.1% for patients with BRCA1 mutation, and 10.2% for BRCA2 mutations [10]. The age of the first cancer is a significant risk factor for contralateral breast cancer. If diagnosed before 41 years, the risk is 23.9% while for patients between 41 and 49 years the risk is 12.6% [11]. Women with BRCA1 mutation are more likely to get triple negative (estrogen receptor, progesterone receptor and HER2 receptor not amplified) tumors, and they are more often higher-grade tumors and basal-like subtype [[12], [13], [14], [15], [16], [17]]. 75% of women with BRCA1 mutation have a triple negative disease, a basal-like phenotype, or both ([18,19]). The subtype and hormone receptor status of a patient with BRCA2 mutation resembles that of sporadic cancer, but it is still associated with worse prognosis [20]. Among patients with BRCA 2 mutation estrogen receptor positive status is associated worse prognosis [20]. In survival studies risk reducing mastectomy (RRM) is associated with lower mortality than surveillance for women with BRCA1 mutation. The same benefit is not seen in women with BRCA2 mutation; hence counseling should be individualized [21]. Interval cancers are those that appear in between screening periods. They are generally more aggressive than screening detected breast cancer. For women with BRCA mutation interval cancers are specifically associated with worse clinicopathologic features and require more aggressive treatment [22]. Mammography and MRI reduce the risk of interval cancers in these high-risk women and should be applied in women who chose surveillance instead of risk reducing surgery [22]. There is a difference in overall survival between breast cancer patients with BRCA1 and BRCA2 mutation. This may be attributed to the more aggressive tumor biology in those with BRCA1 mutation. Disease-free survival is also shorter for women with BRCA1 mutation [23].

This review is meant to address BRCA mutation carriers, both unaffected and affected carriers, with a focus on breast cancer and surgery of the breast. There are many different strategies on how to approach these patients and it is important that they are well informed and prepared for proper surveillance, treatment, and follow-up. The information present in the review is based upon extensive review of the literature with “BRCA” and “surgery” as search terms. More than 1500 publications answered to these search terms. A selection was made on publications covering BRCA and surgery from an oncological breast surgeon's perspective.

### 1.2 Risk of breast cancer in other high penetrance genes

There are other high penetrance gene mutations associated with breast cancer, but BRCA1 and BRCA2 are best elucidated [24]. Other high-penetrance genes include PTEN, TP53, STK11, and CDH1 [25], PALB2 [26], ATM and CHEK2 ([24,27]). Mutation in PALB2 is associated with an increased risk of 35–60% [26], ATM and CHEK2 mutation with a life-time risk of 25–30% [24,27]. Routine for RRM for female with BRCA mutation is more generally accepted than for other high- or moderate risk genes [28]. There are guidelines for management of genetically caused breast cancer with mutations other than BRCA1 and 2, but these are not as rigid as the guidelines for women with BRCA mutation [25]. This review has a focus on BRCA mutation, and the proper surgery associated with this gene mutation. A list of other genes associated with breast cancer is provided in the following webpage (https://www.breastcancer.org/risk/factors/genetics).

### 1.3 Family history of breast cancer without known gene mutation

A specific predisposing gene is identified in <30% of the patients where there is a family history of breast cancer [7]. The biology of a genetically caused breast cancer with BRCA mutation is different from genetically caused breast cancer without BRCA mutation. Those with BRCA mutation are more often hormone receptor negative and more often HER2 negative. The type of surgery performed differs significantly, where female with BRCA mutation more often choose mastectomy, a decision made together with their physician. Risk of recurrence and risk of contralateral breast cancer is the same in genetically caused breast cancer with and without BRCA mutation and it is greater than the risk in sporadic cancers [29].

## 2 Prophylactic bilateral salpingo-oophorectomy (PBSO)

### 2.1 Prophylactic bilateral salpingo-oophorectomy (PBSO) and risk-reduction

Women with BRCA1 mutation are recommended prophylactic bilateral salpingo-oophorectomy (PBSO) at the age between 35 and 40. Women with BRCA2 mutation are recommended the same procedure at a later age, preferably between 40 and 45 [30]. The benefit of this procedure is a reduction of ovarian cancer incidence of 96% and a reduction in breast cancer incidence of up to 50% [31]. Recent studies have however failed to show a significant reduction in breast cancer risk in patient having a PBSO [31]. In women who have already experienced breast cancer PBSO is meant to reduce the risk of contralateral breast cancer, but the significance of this risk reduction is questionable ([32,33]). PBSO further gives a reduction in ovarian cancer specific mortality of 95% and in breast cancer specific mortality of 42% [[34], [35], [36], [37], [38]] The risk reduction in breast cancer is more pronounced for BRCA1 mutation carriers than BRCA2 mutation carriers [34].

### 2.2 Prophylactic bilateral salpingo-oophorectomy and its physical and psychological effect on a young woman

PBSO renders an anticipated early menopause which has unfortunate physical and psychological consequences ([39,40]). Premenopausal women undergoing PBSO experience hot flashes and night sweats (40%), dyspareunia (17%), and decreased libido (22%) [41]. Estrogen is protective against osteoporosis and cardiovascular diseases and is recommended for women with premature menopause in general [42]. Hot flushes are experienced in 80% of women in menopause, and this has great impact on the reduction of quality of life [43]. Hormone replacement therapy (HRT) is the only effective strategy to compensate for the lack of hormones induced surgically. The addition of estrogen alone does not increase the risk of breast cancer in these women but the addition of progesterone, which is needed to protect the endometrium, is not adequately studied to confirm its safety ([30,[44], [45], [46]]). The risk associated with progestins/progesterone may be of little clinical impact in women undergoing both PBSO and RRM [30]. The reduction of breast cancer risk for BRCA positive women with pre-menopause due to PBSO is not affected using HRT([45,47]). In a review with 13 publications there was cumulative evidence to suggest that HRT had positive impact on quality of life, but they claim that the breast cancer risk associated with HRT is still not clear and randomized control trials are needed [48]. The risk of contralateral breast cancer in women with BRCA mutation is reduced in patients adjuvant treated with endocrine therapy, but still, the risk is higher in carriers than non-carriers [33].

## 3 Risk reducing mastectomy and its effect on breast cancer risk

Bilateral risk-reducing mastectomy (RRM) is the most effective approach to reduce risk of breast cancer in patients with genetically caused breast cancer. The expected benefit of prophylactic mastectomy in women with BRCA mutation who have not had their breast cancer (unaffected carriers) differs by age at the time of the procedure being done [49]. The probability of being alive at 80 years old in women having mastectomy at 25 years is increased by 8.7%. The estimated benefit decreases with age and at 50 it is estimated to be 2.8% [49]. The timing of the procedure is crucial, especially in the matter of family planning [50].

The effect of contralateral RRM in women with BRCA mutation diagnosed with breast cancer is debated. There are six studies with similar results all suggesting contralateral RRM has positive impact on survival ([23,[51], [52], [53], [54], [55]]). In one of these studies the effect of contralateral RRM was no longer significant when adjusting for PBSO [55]. A large review addressed this matter and found 61 eligible studies which were all observational studies [56]. They suggested most studies claiming survival advantage in contralateral RRM were biased by healthier and younger patients accounting for the improved outcome. When controlling for confounding factors there was no survival benefit from contralateral RRM [56]. The benefit of contralateral RRM to decrease subsequent contralateral breast cancer is however well documented ([25,51]). Therefore, the contralateral RRM is often preferred by affected women with BRCA mutation to reduce the stress associated with a new cancer.

Women undergoing RRM may experience great discomfort. It has been reported that as many as 69% experience pain after the procedure, up to 71% experience discomfort in the breast, 85% has reported reduced sexual sensation, and enjoyment of sex was reduced in 75% of the patients [57]. These results are from an older study including only 59 women. Measurements for quality of life are more favorable in recent studies [58]. In a systematic review of 22 studies, patients were satisfied with the outcomes and report high psychosocial well-being and positive body image. The sexual well-being and somatosensory function were found to be more compromised [58]. Satisfactions with a reconstructed breast is influenced by realistic outcome expectations, both related to appearance and intimacy concerns [59].

## 4 Who should we test for BRCA mutation and at what time?

The uptake of RRM in unaffected women with BRCA mutation is reported to be between 42 and 54% [60,61] and PBSO is between 52 and 75% [61,62]. Family history of first- and second-degree relatives being deceased from breast cancer was predictive of uptake of RRM and of PBSO [62]. Age and childbirth also influenced the decision ([61,63]). The choice of risk reducing surgery, including RRM and PBSO differs between women with BRCA mutation diagnosed with cancer prior to testing. 61% of the patients with cancer at testing chose bilateral RRM([64]). In any case it is however very important that these women are well informed and discussed in a multidisciplinary team prior to surgery ([65]). There are major decisions to be made by a patient who is found to have a BRCA mutation, and this should be specified to the person prior to testing [66]. There are psychosocial issues to be addressed in relation to testing but women who have been tested do not regret their decision to test even if their test reveals BRCA mutation [67]. Generally it is suggested that the threshold for genetic testing is a 10% likelihood of detecting a mutation based on different risk modelling tools like BOADICEA (https://ccge.medschl.cam.ac.uk/boadicea/), BRCAPRO (https://projects.iq.harvard.edu/bayesmendel/brcapro), Myriad (https://myriad.com/products-services/hereditary-cancers/bracanalysis/), the Tyrer-Cuzick Model Breast Cancer Risk Evaluation Tool (https://ibis.ikonopedia.com/) [68] and the Manchester scoring system (https://www.health-atlas.de/models/2) [69,70]. Indications for increased risk of having a genetic mutation associated with breast cancer is shown in Box 1. There is a cost related to testing and a cost related to the preventive approaches. Therefore, in low-income countries the appropriate counseling is not offered to the patients [71]. It is even shown that there are differences between hospitals within the same region in selecting which patients to test [72]. Patient satisfaction with mastectomy and immediate reconstruction is shown to differ between those undergoing therapeutic mastectomy due to cancer as opposed to unaffected women with BRCA mutation having RRM. Physical well-being is the same but psychosocial and sexual well-being was lower in unaffected BRCA mutation carriers [73].

Box 1 Indicators for a patient to be likely of having a genetic mutation linked to breast cancer.

• Blood relatives on either mother's or father's side who has been diagnosed with breast cancer before the age of 50
• There is both ovarian and breast cancer on the same side of the family or in a single individual
• Family history of triple negative breast cancer
• Family history of other cancer in addition to breast cancer, like prostate, melanoma, pancreatic, stomach, uterine, thyroid, colon, and/or sarcoma
• Family history of bilateral breast cancer
• Patient is Ashkenazi Jewish heritage
• Black race diagnosed with breast cancer at age 35 or younger
• Family history of male breast cancer
• Family history of known genetic aberration in breast cancer related gene

Alt-text: Box 1

## 5 Occult breast cancer on final pathology in a risk-reducing 1

When we speak of occult breast cancer (OBC) we usually refer to clinically recognizable axillary metastatic carcinoma from an undetected breast tumor [74]. Occult breast cancer in the setting of RRM is when there is cancer on final histology of the mastectomy specimen which was not known prior to surgery. To prevent this, pre-surgical mammography and MRI should be performed no later than 6 months prior to surgery [75]. The presence of occult cancer was addressed in a study comparing occult cancer in reduction mammoplasties compared to RRM. The incidence of significant breast pathology was statistically higher in RRM (12.4%) compared to reduction mammoplasties (2.3%) [76], but there was no significant increase in the incidence of malignancy between the two groups (1.2% and 0.6%, respectively) [76]. Occult malignancy in contralateral RRM in a study including women without BRCA mutation was shown to be 6%, and 28% had a high-risk lesion. Multifocality and/or multicentricity in the invasive index cancer was associated with occult malignancy in the contralateral removed breast [77]. Another study of 292 patients had no invasive breast cancer in the RRM, only a few ductal carcinomas in situ (DCIS) (1%), a few lobular carcinomas in situ (LCIS) (1.3%), and a few atypical ductal (1.7%) and lobular hyperplasia (0.3%). There were no positive sentinel lymph node (SLN) in the cases this procedure was performed [78]. In cases of RRM where occult cancer is found on final histology, patients are required to have a complete axillary dissection which is related to risk of arm morbidity [79]. There might be exceptions from the guidelines in selected cases but knowledge of the status of the axillary nodes is required for guidance of adjuvant therapy. The use of SLN in RRM was addressed in a study of 409 patients, 436 RRM, 23 of these patients had BRCA mutation. Occult cancer was identified in 5%, and more than half of these were DCIS. Invasive cancer was found in 1.8% of the mastectomies, none in the BRCA positive patients. Significant increased risk of invasive cancer was seen in postmenopausal patients, patients age above 60 years, and patients history of invasive lobular carcinoma or LCIS [80]. In selected cases one may consider the need for SLN biopsy but not in general [80]. More importantly, occult cancer is not associated with BRCA mutation [80] and should routinely not be indicated ([78,81]).

## 6 BRCA mutation and family planning

### 6.1 BRCA mutation and fertility

In breast cancer patients with BRCA mutations fertility and family planning are important issues since these women are often younger and have more aggressive tumors, which means they will be advised adjuvant or neoadjuvant chemotherapy which potentially reduce fertility through premature ovarian failure ([82,83]). A possible approach to this issue is cryopreservation of the ovaries. A retrospective evaluation of two prospective studies consisting of 156 women where 29 (18.6%) had a BRCA mutation, showed a consistent trend for reduced reproductive potential in BRCA mutated patients measured by anti-Mullerian Hormone (AMH). BRCA1 positive patients had lower AMH level than BRCA2 positive patients. In the process of oocyte cryopreservation BRCA patients needed higher dose of gonadotropins and longer duration of stimulation, and they still retrieved less oocytes than BRCA negative patients [84]. The lower median AMH level and the lower number of retrieved oocytes in BRCA positive patients was not significant (p = 0.109 and p = 0.145 respectively). Poor response rate (retrieval of ≤4 oocytes) was 40.0% in the BRCA positive patients and 11.1% in BRCA negative patients (*P* = 0.147) [84]. Decreased ovarian reserve and poorer response to ovarian stimulation is suggested by others ([85,86]). Women >35 years had ten times the odds of a low AMH compared to women <35 years. After adjusting for body mass index, smoking, gravidity, parity, and age >35 years, BRCA is still significantly associated with a low AMH [86]. More recent studies have shown that women with BRCA mutation with and without malignant disease have the same ovarian reserve and responses to ovarian stimulation compared to BRCA negative cancers and BRCA negative women who are free of cancer ([87,88]). This shows there is conflicting evidence on the impact of BRCA mutation on fertility. The most recent update and systematic review of the literature emphasize the need for individual fertility counseling in BRCA carriers [85].

### 6.2 Pregnancy in breast cancer patients with BRCA mutation

For the general population pregnancy after a breast cancer diagnosis is not associated with inferior outcome compared to matched non-pregnant controls [89]. The safety of pregnancy after breast cancer in women with BRCA mutation is a matter of concern considering their already increased risk of aggressive breast cancer and recurrence of disease. This issue was addressed in a large international, multicenter, hospital-based retrospective cohort study including 1252 women with BRCA mutation, 811 BRCA1, 430 BRCA2, and 11 BRCA1/2 [90]. 195 of these women had at least one pregnancy after breast cancer. Disease free survival and overall survival did not differ between the pregnant women and the non-pregnant women (p = 0.41 and p = 0.66 respectively). Other complications or congenital anomalies in the BRCA positive patients were not more than expected in a cohort of BRCA negative patients, hence pregnancy in women with BRCA mutation after breast cancer could be considered safe both for the mother and the fetus [90]. Fertility protection and treatment involves hormonal treatment which is questionable in high-risk women. Studies on cancer risk of fertility treatment in women with BRCA mutation are few. A systematic review found only 4 studies covering this topic, two of which addressed ovarian cancer and two covering breast cancer risk [91]. The first study on breast cancer risk was a case-control study including 1054 pairs of women with BRCA1 mutation and 326 pairs of women with BRCA2 mutation. They found a possible, but non-significant adverse effect on breast cancer risk in treatment with gonadotropin-containing medication (p = 0.08) which is necessary in fertility preservation [92]. The other study was a prospective cohort study to investigate the effect of letrozole and gonadotropin stimulation for fertility preservation (93). This study included 47 BRCA positive patients where 26 had fertility preservation, and the rest did not. They found no significant difference in relapse-free survival (p = 0.57) or overall survival (p = 0.18) between the two groups [93]. The knowledge and attitude towards fertility and pregnancy of the physicians treating young women with BRCA mutation is variable and should be improved through systematic education [94].

## 7 Surveillance as an alternative to risk reducing surgery in female with BRCA mutation

Surveillance is a reasonable alternative to risk reducing surgery in selected women [95]. Risk reducing surgeries are associated with significant psychological and physical consequences which makes it understandable that some women choose screening with mammography and breast MRI. One study claims that the surveillance with breast MRI and mammography allows detection of breast cancer at an early stage with good survival outcome [96]. A study using anonymous questionnaires reveals that women who follow a surveillance program show a good level of satisfaction because it lowers the concerns of cancer risk. Satisfaction is also high in women undergoing risk reducing procedures mainly for the same reason [40]. The risk reduction benefit of RRM compared to surveillance has not been addressed in randomized studies. It has been addressed prospectively and results show that RRM reduces the risk of breast cancer substantially compared to surveillance but the effect on survival is not as obvious [97]. Surveillance may not be the appropriate choice for all BRCA positive patients, and there are conflicting opinions as to if this is as good as risk reducing surgery. It is debated whether the detection of a breast cancer in a woman choosing surveillance is early enough. Even under tight surveillance the breast cancer diagnosed in BRCA patients are more aggressive and therefore more often require adjuvant chemo- and/or endocrine therapy [98].

## 8 Medical preventive therapies and its effect on breast cancer risk in female with BRCA mutation

Women with BRCA mutation treated for breast cancer have a favorable effect of medical preventive therapy with selective estrogen receptor modulators (SERMs) like tamoxifen and aromatase inhibitors (Ais). This treatment significantly reduces breast cancer risk beyond the active treatment period [99]. Studies have shown that the use of tamoxifen was associated with an approximately 45%–60% reduction in the risk of contralateral breast cancer in affected women with *BRCA1* and *2* mutations ([100,101]). The effect is greatest for BRCA2 mutations, probably because women with BRCA1 mutation more often develop estrogen and progesterone negative cancers. It is also suggested to apply chemoprevention to prevent breast cancer in female with BRCA mutation carriers without prior breast cancer [102], but this is, if necessary, only suggested for female with BRCA2 mutation [103], and it is questionable if it should be advised to these women. There is an increased risk of thromboembolic events and endometrial cancer in patients treated with Tamoxifen [103]. However, women who received Tamoxifen had less fractures caused by osteoporosis, and Tamoxifen had no impact on ischemic heart disease. More importantly, Tamoxifen had no impact breast cancer-related death [103].

## 9 Patients diagnosed with breast cancer are not always aware of having a BRCA mutation

Most patients diagnosed with breast cancer (90%) are not aware of having a BRCA mutation prior to surgery (104). This influence the choice of surgery being performed (p = 0.017) but will not influence the risk of ipsilateral breast cancer recurrence (p = 0.765) or the risk of contralateral breast cancer (p = 0.69) [104]. Patients with preoperative knowledge of BRCA mutation more often have bilateral mastectomy (105). Knowledge of BRCA mutation after initial surgery may lead to a second surgical approach if they initially were treated with breast conserving therapy ([104,105]). In a study of 997 patients evaluating pre- or postoperative genetic testing revealed that 87.2% of BRCA positive patients tested preoperatively underwent bilateral mastectomy. In the group of postoperative tested women 70.6% underwent breast conserving therapy as the first surgical procedure, and 41.2% of these underwent bilateral mastectomy as a second procedure after receiving results from their BRCA-test. They further found that time from diagnosis to first surgery was longer in the preoperative genetic testing group. Predictors of bilateral mastectomy as the first surgical procedure were younger age, bilateral cancer, BRCA1/2-positive results, and preoperative genetic testing [106]. For patients with elevated risk, it is important to be tested before initial surgery for them to make an informed decision together with their physician on which surgical strategy to approach [105]. It is also important to include an experienced specialist in genetics in the decision [107]. Thorough genetic counseling has impact on the decisions made associated with surgery [108]. The choice of having a risk-reducing procedure, either PBSO or RRM, differs between affected and unaffected women with BRCA mutation, the latter more often choose surveillance [109].

## 10 Surgical procedures associated with female with BRCA mutation

### 10.1 Breast conserving therapy in breast cancer patients with BRCA mutation

In sporadic breast cancer breast conserving therapy (BCT) has the same survival data as mastectomy if it is combined with adjuvant radiotherapy ([110,111]). Some studies have even demonstrated better survival data in patients treated with BCT ([112,113]). There is however a certain risk of ipsilateral breast cancer recurrence in patients treated with BCT. In a study including 445 patients with sporadic breast cancer, the estimated 10- and 15-years of ipsilateral recurrence was 9% and 17%, respectively (33). Patients with BRCA mutation treated with BCT have, in multiple studies, shown to have the same risk of ipsilateral recurrence as those with sporadic breast cancer (12% in 10 years and 24% in 15 years) [33,[114], [115], [116], [117], [118], [119]]. Literature is controversial concerning the long-term local outcome ([120,121]). In studies with long time follow-up there have been demonstrated an increased risk of ipsilateral breast cancer in women with BRCA mutation compared to women with sporadic breast cancer, but it is not associated with adverse short- and longtime survival outcome ([118,122]). Due to the increased risk of ipsilateral breast cancer in women with BRCA mutation it has been argued that these women are not suited for BCT. This issue has been addressed in studies comparing BCT to mastectomy in breast cancer patients with BRCA mutation ([123,124]). Results show that there is an increased risk of ipsilateral recurrence in those treated with BCT, but it has no impact on overall survival (124). It has been argued that most ipsilateral events are new primary cancers [125]. There is however an increased risk of contralateral breast cancer in women with BRCA mutation [33,[115], [116], [117],^126^,^127^]. The risk is estimated to 25–30% over 10 years and more than 40% over 15 years ([33,126]). The above-mentioned risk of ipsilateral breast cancers in BRCA positive patients is lower than that of contralateral breast cancer and is probably an effect of the irradiation of the breast being treated as part of the breast conserving therapy ([33,126]). Because of the risk reducing effect of PBSO on the contralateral breast claimed in some publications (this is controversial [32,33]), it is suggested that woman who have not had PBSO should be offered mastectomy instead of BCT ([32,33]).

BCT requires adjuvant radiotherapy to reduce risk of local recurrence. There are different opinions on the safety of adjuvant radiation therapy and the risk of contralateral breast cancer. BRCA mutation involves mutation and malfunction of the DNA damage repair pathways. DNA damage is the same mechanism applied when treating the patient with adjuvant radiotherapy. Therefore, it has been argued that there is an increased risk of radiation induced contralateral breast cancer. This was evaluated in a study of 247 patients who experienced contralateral breast cancer, 169 received radiotherapy and 78 did not. Conclusion from this study was that compared to patients with sporadic breast cancer women with BRCA mutation had an increased risk of developing a radiation induced contralateral breast cancer ([128]). Others have concluded the opposite, namely that there is no evidence of increased toxicity or contralateral breast cancer from radiation exposure in female with BRCA mutation ([129,130]). The increased risk of contralateral breast cancer in women with BRCA mutation is not a cause of the radiotherapy, but the results of the already increased risk of a contralateral breast cancer [124]. One study found that irradiation of the contralateral breast in breast cancer patients with BRCA mutation reduced the incidence of contralateral breast cancer [131]. Overall young patients, patients with triple negative disease and those with BRCA1 or 2 mutations have the highest risk of local failure. It is considered that the intrinsic biology is the reason for this inferior outcome, and this is not compensated for by bigger surgery like mastectomy [132].

Conclusively BCT is not contraindicated in female with BRCA mutation who experience primary breast cancer, however ipsilateral therapeutic mastectomy and contralateral RRM should be discussed with the patient considering the increased risk of contralateral breast cancer compared to those with sporadic cancers, and that there is a risk of ipsilateral primary event even though the latter does not seem to be different between women with or without BRCA mutation [25]. All the studies claiming the safety of BCT are observational studies with the known risk of selection bias. Longer follow-up studies are awaited with interest [25].

### 10.2 When to perform a risk reducing mastectomy and which surgical procedure should be performed

There are different approaches to prophylactic surgical procedure both when it comes to time of surgery, number of surgeries, the order of the different surgical procedure, and how the surgical procedures should be performed. The approach to the surgical strategy differs between unaffected and affected women with BRCA. In BRCA carriers not diagnosed with breast cancer a combined approach with concurrent PBSO and bilateral prophylactic mastectomy (RRM) is preferred [133]. The combined approach may however be offered to patients who have been diagnosed with cancers as well [134], but it requires good planning. BRCA related cancers are often aggressive, and it is important not to delay treatment as this may compromise prognosis. For women with breast cancer and BRCA mutation scheduled for therapeutic mastectomy on the affected side, contralateral RRM should be considered [25].

**Fig. 1:**
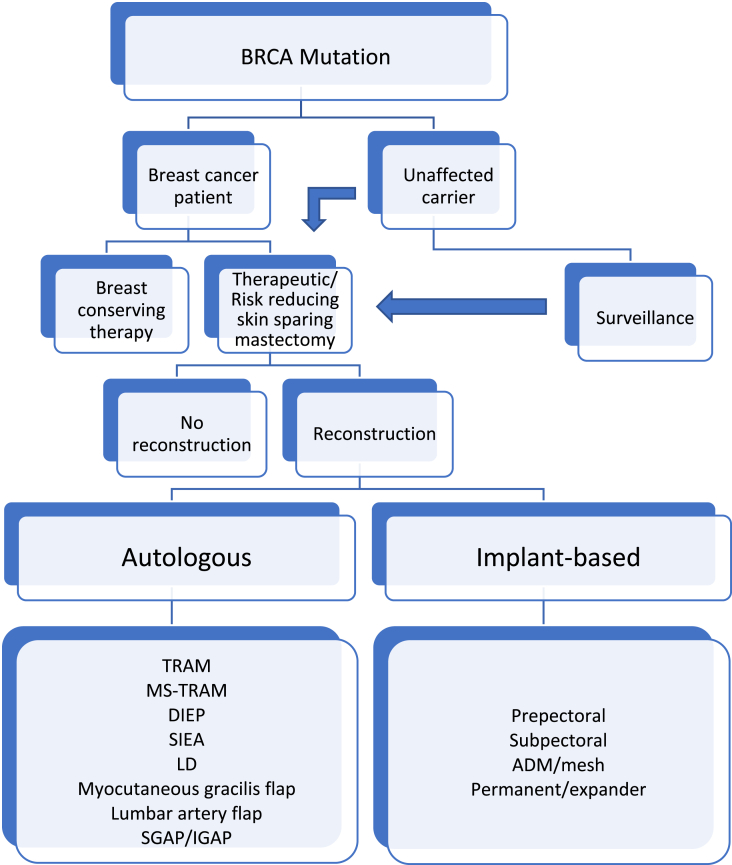
Management of patients with BRCA mutation. Surveillance is often combined with later surgery where timing is according to family planning. ADM – acellular dermal matrix. TRAM – trans abdominis myocutaneous flap. MS-TRAM – muscle sparing TRAM. DIEP – deep inferior epigastric artery perforator flap. SIEA – superior inferior epigastric artery perforator flap. LD – latissimus dorsi flap. SGAP – superior gluteal artery perforator flap. IGAP – inferior gluteal artery perforator flap.

Fig. 1 is an example on the algorithm in management of both the affected and the unaffected BRCA carriers. In some of the steps it is possible to change course, that is one can choose surveillance initially and when time is ready the patient can perform risk reducing surgery. This is often dependent on age and family planning.

#### 10.2.1 Implant-based breast reconstruction

**Fig. 2:**
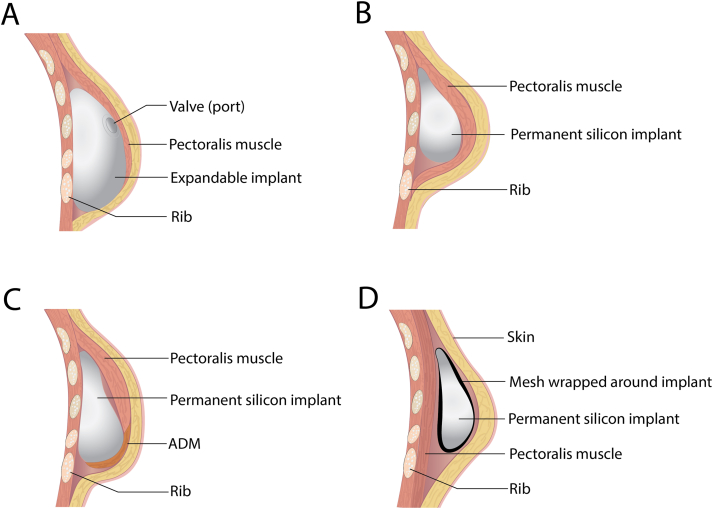
Implant-based reconstruction. A and B) Traditional two-stage expander implant reconstruction. A) tissue expander placed in a subpectoral pocket. The expander can successively be expanded through a needle injected transcutaneous into a Valve/port which is palpable through the skin above the implant. B) Exchange of the tissue expander to permanent silicone implant. C) Single stage submuscular implant reconstruction with mesh/ADM. D) Single-stage subcutaneous implant-based reconstruction with mesh wrapped around the implant.

RRM can be performed both with and without primary reconstruction. There is an increased risk of complications and a possible second surgical procedure in patients having primary reconstruction compared to a simple mastectomy [135]. Immediate reconstruction is still preferred for psychological reasons. The most common procedure is the RRM with an implant-based reconstruction (Fig. 2). This can be performed in several ways. The implant can be permanent (Fig. 2 C, D) or a tissue-expander which is changed into a permanent silicone implant in a second surgical procedure (Fig. 2A and B). It can be placed pre-pectoral (subcutaneous) or subpectoral (Fig. 2), and it can be with or without preservation of the nipple. All these are details to be discussed with the patient prior to surgery and some of the final decisions may be performed during surgery. Preoperative evaluation of comorbidities like uncontrolled diabetes mellitus, morbid obesity, tobacco use, history of radiation, subcutaneous tissue thickness, and availability of possible donor site for fat grafting are important aspects in deciding the proper location of the implant and which implant to use. From an oncological aspect it is mandatory to evaluate the distance of the tumor to the chest wall, and the stage of the tumor. Patients with skin innervation and muscle innervation are mainly excluded from having a primary reconstruction [75]. The implant-based technique is application of an immediate implant (permanent implant) or a tissue expander. The surgical team should be prepared for and have both options available, and the final decision should be made during surgery. Other intraoperative considerations are the quality of the skin flap after the skin sparing mastectomy, and if the dermal edges are well circulated. There should be adequate amount of dermis, and there should not be tension in the skin flap upon closure [136].

An example of an implant-based approach is the nipple-sparing bilateral prophylactic mastectomy and immediate reconstruction with TiLoop®Bra mesh. This is a procedure where the implant, which is covered by a mesh, is placed subcutaneously (Fig. 2D), and is found to give high levels of satisfaction and quality of life ([137,138]). An alternative approach to support the implant is to use an acellular dermal matrix (ADM) to support the lower pole of the implant (Fig. 2C) [139]. There is a lack of good quality studies to support the safety of ADM in immediate reconstruction [139].

#### 10.2.2 Autologous flap-based reconstruction

**Fig. 3:**
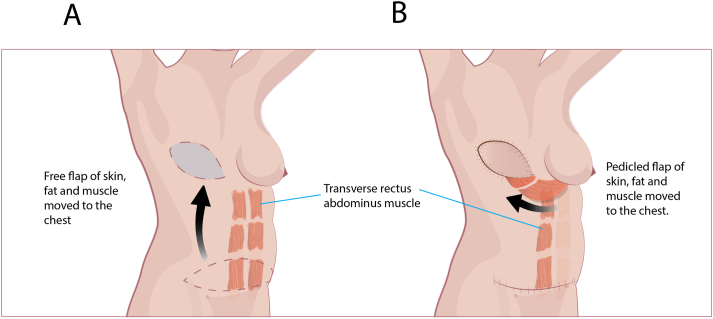
Trans rectus abdominis myocutaneous flap (TRAM) is the most common technique. It can be done with a free flap (A) or pedicled flap (B). It can also be performed by a muscle-sparing trans rectus abdominins myocutaneous flap (MS-TRAM). In a pedicled TRAM flap fat, skin, blood vessels, and muscle from the lower abdominal wall is transferred under the skin up to the chest to rebuild the breast. The blood vessels of the flap are left attached to their original blood supply in the abdomen. In the free flap a flap of skin, fat, and all or part of the underlying rectus muscle are used to reconstruct the breast. Blood vessels of the flap is reattached to blood vessels on the chest using microsurgery.

**Fig. 4:**
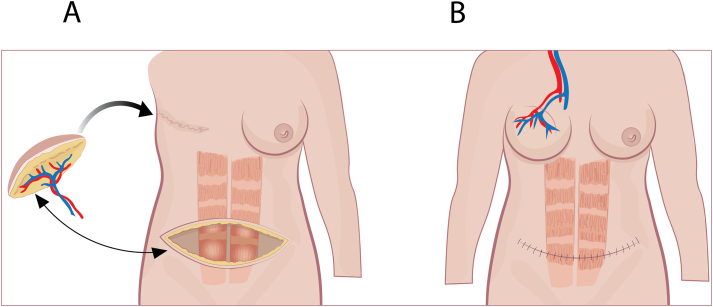
An example of autologous breast reconstruction using the DIEP technique. Deep inferior epigastric perforator (DIEP) flaps and superior inferior epigastric artery (SIEA) flap are named after which artery is donated to supply the transplant. DIEP is similar to a MS-TRAM flap except that no muscle is used to reconstruct the breast. In a DIEP flap, fat, skin, and blood vessels are donated from the lower wall of the abdomen (A) and moved to the chest wall to reconstruct the breast. The artery of the flap is connected to the arterial supply of the chest (B).

As an alternative to implant-based reconstruction, the reconstruction can be done based on autologous tissue. Trans rectus abdominis myocutaneous flap (TRAM) is the most common technique [140] (Fig. 3). It can be done with a free flap (Fig. 3A) or pedicled flap (Fig. 3B). It can also be performed by a muscle-sparing trans rectus abdominins myocutaneous flap (MS-TRAM). In a pedicled TRAM flap fat, skin, blood vessels, and muscle from the lower abdominal wall is transferred under the skin up to the chest as a replacement for the removed breast. The blood vessels of the flap are left attached to their original blood supply in the abdomen (Fig. 3B) [141,142]. In the reconstruction based on a free flap the removed breast is replaced by a flap of skin, fat, and all or part of the underlying rectus muscle. Blood vessels of the flap is reattached to blood vessels on the chest using microsurgery (Fig. 3A). The name of the procedure is based on which artery is donated to supply the transplant. The most common are deep inferior epigastric perforator (DIEP) flap [141] (Fig. 4) and superior inferior epigastric artery (SIEA) flap [143]. DIEP is like a MS-TRAM flap but without the muscle from the lower abdominal wall as part of the reconstruction. In a DIEP flap fat, skin, and blood vessels are donated from the lower wall of the abdomen and moved to the chest wall to reconstruct the breast. Blood vessels of the flap is reattached to blood vessels on the chest in a similar manner as the free TRAM flap (Fig. 4) [141].

Indications for autologous based reconstruction over implant-based reconstruction is above all patient preference. It is more often used in unilateral reconstructions because of the amount of abdominal tissue needed to reconstruct both breasts. Therefore, it might be a challenge in BRCA mutation carriers who need bilateral reconstruction. A woman with enough abdominal bulk is more suited for this procedure but obesity is a relative contraindication. Other relative contraindications are prior abdominal surgery, prior abdominal contouring procedures like liposuction and abdominoplasty, smoking history less than a month before surgery, and if the patients suffer from a hypercoagulable state [142]. There are also absolute contraindications to autologous reconstruction and that is above all if the patient is medically unfit. Significant abdominal surgery affecting the vasculature is an absolute contraindication, and if the procedure gives a significant delay in treatment of a disease in case of an affected female with BRCA mutation [142]. It is important that the patient has realistic expectation of results [59].

There are alternative donor sites for both pedicle and free flap. Latissimus dorsal pedicle flap is an option, but this procedure is often combined with an implant-based reconstruction or a breast conserving therapy to fill the defect breast volume [144]. The latter is more applicable to BRCA negative women since they more often have breast conserving therapy. Free flap from the abdomen requires a certain volume which is not always present in young women with BRCA mutation. An alternative is the lumbar artery perforator flap which is possible also in very thin patients ([145,146]). The transverse myocutaneous gracilis free flap is a third alternative, and this procedure is more suitable for women with small and medium sized breasts and not enough abdominal bulk for a regular DIEP [147]. Lastly there is a possibility to use the gluteal region as donor site with the inferior or superior gluteal artery perforator flap. It may be technically challenging but in the hands of experienced microsurgeons it is consider safe and reliable with nice aesthetic outcome and minimal donor-site morbidity [148]. RRM and reconstruction with autologous tissue is more often performed in unaffected female with BRCA mutation since it requires more effort both from the patient and from the surgical team, and decisions regarding this procedure are more time consuming and should be well planned. It is however preferred in patients with a prior breast cancer diagnosis treated with adjuvant radiation to the chest wall, due to the increased complication rate associated with post-radiation and implant-based reconstruction [149].

A single institutional study of 238 patients compared implant-based versus autologous reconstruction, 89.5% had implant-based and the rest had autologous based. Women who underwent autologous based reconstruction were more satisfied than those who had implant-based reconstruction [150]. This is also concluded in a recent meta-analysis of 9 studies with more than 2000 implant-based reconstructions and approximately 1000 autologous breast reconstructions [151]. There was significantly higher satisfaction with the breast, higher satisfaction of outcome, higher psychosocial well-being, and higher sexual well-being in autologous reconstruction versus implant-based reconstruction. There was a trend towards better physical well-being, but this was not significant [151].

In cases where the PBSO is combined with the bilateral RRM and primary reconstruction based on autologous tissue it is important to be well prepared. If the PBSO is performed through a laparoscopic technique, it is important to place the ports where they will not interfere with the donor site. PBSO is then performed as the first part of the procedure followed by bilateral RRM and breast reconstruction with for instance DIEP (deep inferior epigastric perforator flaps). This one-step procedure is shown to be safe, feasible and well accepted [133]. The combined approach is positive in terms of cost-effectiveness ([152,153]), particularly for patients who had prior radiotherapy. Free flap surgery is an excellent option for autologous breast reconstruction, with low rate of donor site morbidity and low complication rate [153].

Remodeling of a breast by autologous fat grafting (lipofilling) is a popular technique for breast reconstruction but its oncological safety is debated [154]. It was evaluated in a systematic review including 23 publications. Conclusively they found the technique to be oncological safe and with low morbidity in women with prior breast cancer [154]. A more recent review of 35 studies confirm that fat grafting is a useful reconstructive tool, with good cosmetic outcome and acceptable complication rate [155]. None of these studies addressed BRCA mutation patients specifically. The authors stress the urgent need of randomized controlled trials and long-term follow-up. In high risk patients like women with BRCA mutation, fat-grafting has been shown to give adverse oncological outcome [156] and is generally not advised [75].

#### 10.2.3 Preservation of the nipple

Preservation of the nipple is positive correlated with psychosocial and sexual well-being [157]. The safety of nipple sparing mastectomy (NSM) in sporadic cancer is well documented [158] but its application in female with BRCA mutation is a matter of debate [159]. There are limited published data specific for NSM in breast cancer patients with BRCA mutation. Two studies with 26 and 51 patients respectively, addressed this question specifically and concluded that nipple-sparing mastectomy in appropriately selected women with BRCA1 or BRCA2 mutation is associated with low rates of locoregional recurrence and low complication rates. Larger series and longer follow-up are urgently needed ([160,161]). If the nipple can safely be preserved from an oncological point of view, NSM is preferred. Preservation of the nipple is also possible for women with ptosis, but this requires a certain surgical technique [162]. NSM in a contralateral RRM is also considered safe and should be the appropriate choice for BRCA positive breast cancer patients having a therapeutic NSM on the affected side [163]. It is important to inform the patient that breast tissue will be left behind both for NSM and skin sparing mastectomy with excision of the nipple and the risk of breast cancer is therefore not eliminated.

## 11 Planning of surgical treatment and information to the patients

### 11.1 Good planning and the influence of a multidisciplinary team

Prophylactic mastectomy and primary reconstruction require good planning, both in cancer patients and in non-affected high-risk patients. It is shown that a multidisciplinary model of trained and committed members is favorable for timing and outcome of surgery [164]. A nipple-sparing mastectomy and immediate breast reconstruction should be performed between 30 and 40 years, and a multidisciplinary team should be involved [165]. The women must be well informed about the possible complications, distress, and morbidity associated with a risk reducing procedure.

Surgical management and prevention of breast cancer is under continuous change and improvement. It is important to at all time have trained personnel and well-qualified centers [[166], [167], [168]].

### 11.2 Information on complications to surgery

RRM is the most powerful prevention of breast cancer in BRCA mutation carriers, with a 90–95% reduction rate [169,170]. The risk is not eliminated. Even though there is a small chance of developing breast cancer after RRM, patient should be informed of the possibility. This is especially important in young women who chose RRM prior to pregnancy. In later pregnancies they will most probably experience growing tissue or lumps on the chest wall as a hormonal response to the pregnancy. These changes are to be expected since all breast tissue will not be removed by the mastectomy.

Complications vary according to type of surgery, but there are as with all type of surgery, patient related factors like diabetes, body mass index and smoking [171]. There are certain elements the surgeon must consider and enlighten the patient about. The first is that the risk of breast cancer is not eliminated completely. There is still a 5% risk of getting breast cancer since there will always be unperceived breast tissue left behind. The second important issue is to inform the patient on the increased risk of surgical morbidity like ischemia of the skin or nipple-areolar complex, hematomas, infections, implant failure or autologous flap loss. The surgical morbidity rates of RRM is 15–20% [172].

There are online tools which are shown to be of great value for patients in the decision of which reconstruction to choose (https://www.bcna.org.au/resource/breconda). This is valid for all patients in need of a reconstruction, not just women with BRCA mutation [173].

## 12 Male breast cancer and BRCA mutation

Male breast cancer is rare, and they are more often BRCA mutation carriers compared to women, preferably BRCA2 mutations ([174,175]). In a publication including 49 men with breast cancer, 18.4% had a BRCA mutation [176]. Therefore it is recommended to discuss genetic testing in all men with breast cancer and if necessary refer them to a specialist in cancer genetics [177]. The cumulative incidence of breast cancer at the age of 70 years in men is 1% for BRCA1 mutation and 7% in BRCA2 mutation [178]. The lifetime risk of breast cancer in the general male population is 0.1% [179]. Men with BRCA mutation have a lower survival than men with wild-type BRCA [174]. Surgical treatment of men with breast cancer whether they have a BRCA mutation or not, is based upon the same principle as treatment for women. There is however a concern that there are no randomized trials of surgical treatment with male breast cancer. The approach to treatment and follow up is extrapolated from studies including women and where men often are excluded. BCT is considered as safe in men as in women, but data from large registries show that only 18% of men with tumors less than 2 cm undergo BCT [180]. Most men undergo mastectomy but the principle of surgery in the axilla is the same as in women [181]. There is not much work done to describe the issue of prophylactic surgery in men with BRCA mutation.

## 13 Surgical procedures according to age

Age is no limit for surgery. In a retrospective study where patients over 70 years were tested and found to be BRCA mutation carriers, 16% of patients without prior cancer had a risk-reducing mastectomy [182].

## 14 Risk reducing procedures in women without BRCA mutation

Another important question is if women without BRCA mutations should be advised or accepted for RRM if they request the procedure. Bilateral mastectomy will not provide any survival benefit and is associated with worsened body image and quality of life. In addition there are more re-do surgeries and more complications compared to breast conserving therapy on the affected side [183]. The higher risk of contralateral breast cancer in female with BRCA mutation justifies the choice of contralateral RRM in these patients, but the same benefit is less evident in those without mutations, even though there is a strong family history [184].

## 15 The cost-effectiveness of a risk reducing surgical procedure and which patients should be offered testing

Evaluation of the cost-effectiveness is challenging considering the psychological stress of experiencing a breast cancer diagnosis. It has been suggested that the long-term health-care costs can be reduced by performance of risk-reducing surgeries in BRCA positive patients, both PBSO and RRM([185,186]). Guidelines for whom to test for BRCA mutation varies between institutions. Current guidelines fail to identify all patients with BRCA mutation in a breast cancer population [187]. A study demonstrating the net health-care cost measured in Euro per life-year gained suggests all patients with breast cancer should be tested and this was superior to focus on family history [188].

## 16 Future perspectives in the management of patients with BRCA mutation

Molecular profiling of breast cancer initiated in the millennium change, revealed the heterogeneity of breast cancer, and separated it into molecular subtypes of specific clinical implication including different prognosis and different response to treatment [[189], [190], [191]]. This has directed breast cancer therapy to be more personalized both oncological and surgically. This is an important argument in favor of testing for BRCA mutation, and other cancer related genes, in breast cancer patients also in the absence of a family history of breast cancer. The possible BRCA mutation will, as this review has elucidated, have great impact on the surgical procedure but also on the choice of medical treatment. Poly ADP-ribose polymerases (PARPs) act through base excision repair pathway for single stranded DNA breaks. Inhibitors of the PARP enzyme will therefore block the mechanism of DNA repair. Since patients with BRCA mutation have an impaired DNA repair mechanism, PARP inhibitors are effective in the treatment of these patients ([192,193]). The same principle is the increased sensitivity to DNA damaging chemotherapy, including platinum agents and more resistance to microtubule agents, like taxanes [[194], [195], [196]]. Conclusively there are many arguments in favor of doing genetic testing and genetic counseling, but this requires having a well-educated and qualified multidisciplinary team available to meet these patients and give them the proper medical and surgical treatment.

## 17 Summary

There are guidelines as to which patients to test for BRCA mutation and when is the appropriate timing. It is important that the patients are offered genetic counseling parallel to the testing. Surveillance with breast MRI and mammography may be a good option for some patients. RRM should be discussed with the patient and the surgical method should be discussed in a multidisciplinary team prior to the discussion with the patient. It is mandatory that the choice of surgical procedure is based upon informed consent from the patient. BCT is safe for BRCA positive patients who are diagnosed with breast cancer, and this may be the best immediate option. A RRM can safely be performed later. If the woman is prepared and surgery will not delay further treatment of the breast cancer, a bilateral mastectomy may be suggested, therapeutic on the affected side and prophylactic RRM on the contralateral side. Bilateral RRM is a good option for unaffected women with BRCA mutation. NSM is considered safe and should be offered to the patient if it is technically possible. In a BRCA mutation carrier diagnosed with breast cancer the choice of surgery should be influenced by the age of the woman and her situation according to family planning.

## Ethical approval

The review does not need ethical approval since it is a review of the literature and individual patients are not mentioned.

## Sources of funding

The author has no funding for this review. She is employed in a surgical department and have written the manuscript parallel to her clinical work.

## Author contribution

The author has written the manuscript by herself. The head of the department has read through the manuscript but have not made any changes.

## Trial registry number

Name of the registry:
Unique Identifying number or registration ID:
Hyperlink to your specific registration (must be publicly accessible and will be checked):

## Consent

Not relevant for this review.

## Guarantor

The author takes full responsibility for the content of the manuscript.

## Provenance and peer review

Not commissioned, externally peer reviewed.

## Authors agreement

The author has written the manuscript alone.

## Declaration of competing interest

The author confirms no conflict of interest.
